# Early childhood undernutrition, preadolescent physical growth, and cognitive achievement in India: A population-based cohort study

**DOI:** 10.1371/journal.pmed.1003838

**Published:** 2021-10-27

**Authors:** Apurv Soni, Nisha Fahey, Zulfiqar A. Bhutta, Wenjun Li, Jean A. Frazier, Tiffany Moore Simas, Somashekhar M. Nimbalkar, Jeroan J. Allison

**Affiliations:** 1 Department of Medicine, UMass Chan Medical School, Worcester, Massachusetts, United States of America; 2 Department of Population and Quantitative Health Sciences, UMass Chan Medical School, Worcester, Massachusetts, United States of America; 3 Department of Pediatrics, UMass Chan Medical School Worcester, Massachusetts, United States of America; 4 Centre of Excellence in Women and Child Health, Aga Khan University, Karachi, Pakistan; 5 Centre for Global Child Health, The Hospital for Sick Children, Toronto, Canada; 6 Department of Psychiatry, UMass Chan Medical School, Worcester, Massachusetts, United States of America; 7 Department of Obstetrics and Gynecology, UMass Chan Medical School, Worcester, Massachusetts, United States of America; 8 Department of Pediatrics, Bhaikaka University, Karamsad, Gujarat, India; Emory University, UNITED STATES

## Abstract

**Background:**

There is a lack of nationally representative estimates for the consequences of early childhood undernutrition on preadolescent outcomes in India. Understanding this relationship is helpful to develop interventions that not only prevent child undernutrition but also mitigate its consequences.

**Methods and findings:**

In this cohort study, we analyzed prospectively gathered data from 2 waves of the India Human Development Survey (IHDS) to investigate the association of undernutrition during early childhood (0 to 5 years) in 2004 to 2005 with physical and cognitive outcomes during preadolescent (8 to 11 years) years in 2011 to 2012. These surveys interviewed 41,554 households across all 33 states and union territories in India in 2004 to 2005 and reinterviewed 83% of the households in 2011 to 2012. Primary exposure was assessed using the Composite Index of Anthropometric Failure (CIAF) based on 2004 to 2005 survey. Primary outcomes were short stature (height-for-age z-score [HAZ] <−2), thinness (body mass index [BMI] <18.5 kg/m^2^), reading, and arithmetic skills during preadolescence based on the 2011 to 2012 survey. Survey-weighted generalized linear models were used, and effect modification based on child sex and sociodemographic variables were evaluated using 3-way interaction terms. Of the 7,868 children included in this analysis, 4,334 (57.3%) were undernourished. Being undernourished was associated with increased odds of short stature (odds ratio [OR] 1.73, 95% confidence interval [CI] 1.45 to 2.06) and thinness (OR 1.52, 95% CI 1.33 to 1.73) during the preadolescent period, while it was associated with decreased odds of achieving a higher reading (cumulative odds ratio [cumOR]: 0.76, 0.66 to 0.87) and arithmetic (cumOR: 0.72, 0.63 to 0.82) outcomes. The disparity in outcomes based on CIAF increased with age, especially for female children. Increased level of female education within the household reduced the disadvantages of undernutrition among female children. Study limitations include observational and missing data, which limit our ability to draw strong causal inferences.

**Conclusions:**

In this study, we found that early child undernutrition was associated with several adverse preadolescent physical and cognitive outcomes, especially among female children. Improved female education mitigates this association. Female education promotion should assume a central role in Indian public health policy making.

## Abstract 2

Apurv Soni and co-workers study child nutrition and developmental outcomes in India.

## Author summary

**Why was this study done?:**

- India has the largest number of undernourished children under the age of 5.
- Previous studies report an absence of gender disparities among child undernutrition in India despite well-documented disparities in adulthood.
- Current national programs for addressing child undernutrition focus on the first 5 years of life and do not include gender-focused interventions.

**What did the researchers do and find?:**

- We carried out a population-based cohort study based on data gathered prospectively from nationally representative survey of 41,554 households in India in 2004 to 2005 and a follow-up in 2011 to 2012 among 83% of the same households.
- This study leverages India’s only nationally representative panel dataset to report that undernutrition in the first 5 years of life is associated with increased odds of adverse physical outcomes, i.e., having short stature and being very thin and cognitive outcomes, i.e., unable to read and do arithmetic during preadolescent years.
- Female children who were undernourished were the most vulnerable for experiencing these adverse outcomes, and the gender disparities widened with age during the preadolescent period. As an example, the adjusted probability of short stature for undernourished female children increased from 31% among 8 year olds to 45% among 11 year olds. There was no such increase observed among male children.
- Presence of a woman in the household with more than secondary level of education mitigated the disadvantage associated with both being undernourished and being a female child.

**What do these findings mean?:**

- Sex differences in physical and cognitive outcomes emerge during the preadolescent period and are associated with early childhood nutrition.
- Higher level of female education within the household is an important protective factor for these disparities.
- Elevation of women’s status through improved female education should be central to the national level programs in India focused on maternal and child health.

## Background

India has the largest number of undernourished children worldwide [1]. Most national programs and initiatives in India have focused on the early childhood period, i.e., first 5 years of life [2–4]. There is limited empirical evidence of the consequences of early childhood undernutrition in India [5]. Understanding how early childhood undernutrition affects adolescent health and outcomes is important to inform effective public health strategies.

Despite the widely documented preference for male children in India [6], nationally representative data from multiple sources have found that male children are more likely to experience undernutrition [7,8]. However, adult Indian men are considerably taller than Indian women, and the height difference between genders is of greater magnitude than western countries [9]. Similarly, Indian men have higher social and economic capital than Indian women [10]. Understanding how the disadvantage for women emerges in the setting of apparent nutritional advantage in early childhood period can be useful to guide strategies that enhance gender equality in India.

We used data from India’s first nationally representative panel dataset to investigate the association of undernutrition during early childhood (0 to 5 years) with physical growth and cognitive achievement during the preadolescent period (8 to 11 years). We further assessed whether the observed associations differed by sex and other sociodemographic characteristics.

## Methods

### Data source and sample

**Fig 1:**
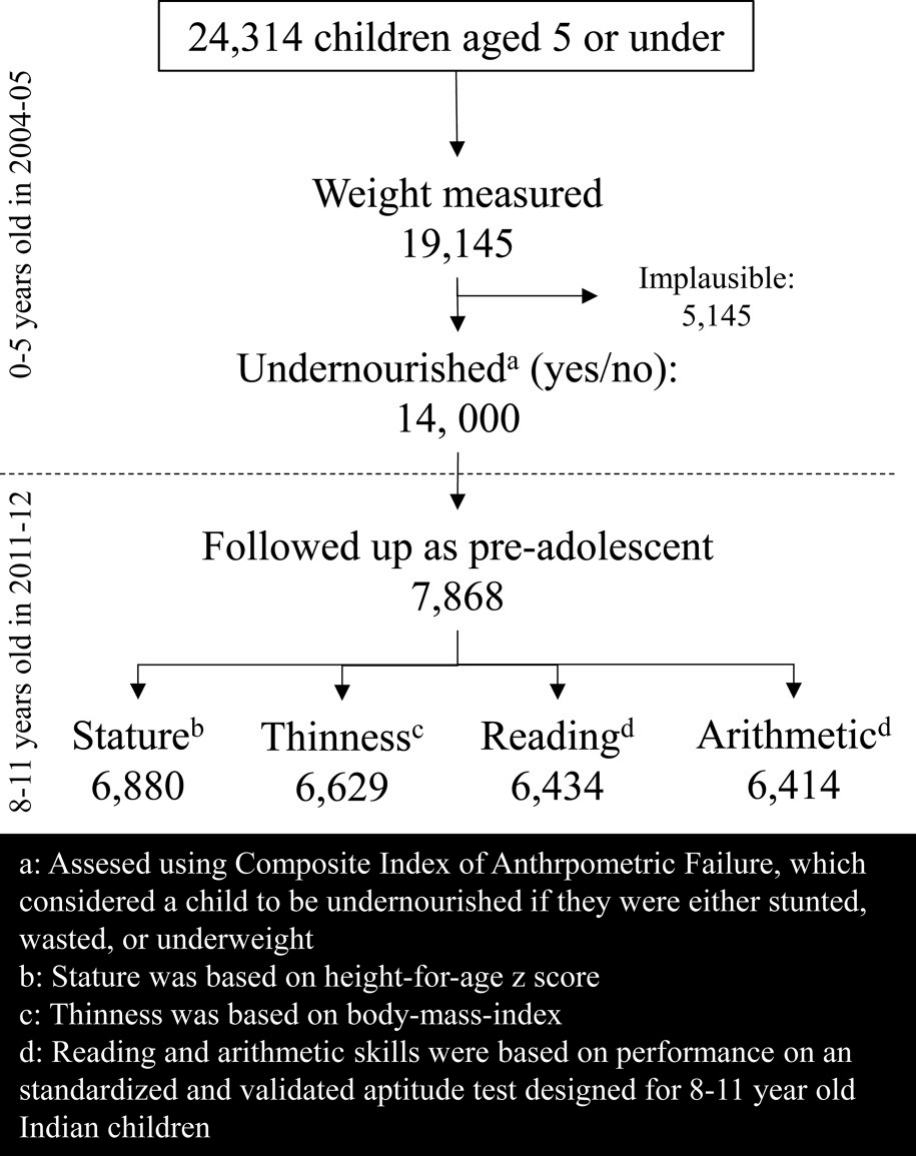
Flowchart demonstrating analytic sample based on children from the IHDS. IHDS, India Human Development Survey.

This cohort study was based on an analysis of prospectively gathered, publicly available data from the 2 waves of the nationally representative surveys of the India Human Development Survey (IHDS-I: 2004 to 2005 and IHDS-II: 2011 to 2012) [11,12]. The IHDS studies were conducted through a collaboration between researchers from the University of Maryland and the National Council of Applied Economic Research in New Delhi. A detailed description of the survey methodology, including information about data collection, funding, and quality assurance, has been previously documented [11,12]. In brief, IHDS-I surveyed 41,554 households from 33 states and union territories, which were identified using a random stratified sampling method for urban and rural settings. IHDS-II surveyed 42,152 households, including 83% of households that were interviewed during IHDS-I. The data are available through the Inter-university Consortium for Political and Social Research (ICPSR) to its members [11,12]. We restricted the present study sample to children who were under the age of 5 years in 2004 to 2005 and were reinterviewed when they were 8 to 11 years of age in 2011 to 2012 (**Fig 1**). The weighted distribution of participants’ characteristics did not differ substantially between the overall sample of children and those that were included in the analytical sample (**S1 Table**). The study findings are reported according to the REporting of studies Conducted using Observational Routinely collected Data (RECORD) statement (see **S1 RECORD Checklist**).

### Exposure

Undernutrition during early childhood (0 to 5 years) was assessed using the Composite Index of Anthropometric Failure (CIAF) because it is a more comprehensive indicator for the overall burden of child undernutrition in a population [13]. Sex-adjusted z-scores for height-for-age z-score (HAZ), weight-for-age z-score (WAZ), and weight-for-height z-score (WHZ) were calculated using the *zanthro* command in Stata for data of children surveyed in IHDS-I during 2004 to 2005 using the World Health Organization’s (WHO) growth reference curves [14]. Children with either a HAZ, WAZ, or WHZ score below −2 were considered to be positive for CIAF [13].

### Study outcomes

We used IHDS-II data from 8- to 11-year-old children to construct our physical and cognitive outcome variables. We considered 2 indicators of physical growth during the preadolescent period (8 to 11 years): HAZ and body mass index (BMI). We dichotomized HAZ as short stature (z-score below −2) or normal stature (z-score of −2 or higher) and BMI as thin (<18.5 kg/m^2^) and normal (18.5 kg/m^2^ or higher). Cognitive achievement was measured using standardized tests [15]. The tests measured reading ability, categorized as a 5-level variable: cannot read, reads letters, reads words, reads paragraphs, and read stories. Arithmetic ability was also assessed at 4 levels: does not recognize numbers, recognizes numbers, can subtract, and can divide. The tests were administered in all 12 major languages of India, and the interviewers were trained by the Pratham nongovernmental organization to administer the assessments using techniques employed by them for Annual Status of Education Report (ASER) surveys [15]. We considered the cognitive achievement outcomes separately as ordinal variables and dichotomized them to facilitate comparison across literature: can read paragraphs (yes or no) and can subtract (yes or no).

### Effect modifiers and potential confounding variables

We investigated the differential relationships of early childhood nutrition and preadolescent outcomes among male and female children across different subpopulations. Although the underlying construct behind this investigation focused on the child’s gender, the operational definition of the variable was derived from maternal reports of the child’s sex in IHDS-I. Rural and urban location was determined based on the census classification of the region in IHDS-I, while household caste was based on self-report by the head of the household and categorized as General, Other Backwards Class, or Scheduled Caste/Tribe. We categorized monthly expenditure into quartiles (1 = lowest and 4 = highest) because previous investigations using the IHDS datasets have used this metric to characterize household wealth since sources of income varies by occupation and setting across India [12]. We operationalized education as 2 separate variables: highest male and female education in the household [0 = none, 1 = primary (1 to 5 years), 2 = secondary (>5 to 10 years), and 3 = > secondary (10+ years)]. Our decision to consider highest male and female education level within the household was based on the cultural context of households in India, where joint family structures are favored and there are multiple sources of influences for decision-making [16]. We also considered household size, religion, type of school, child’s age in 2012, age at school enrollment, years of schooling, and absenteeism from school as potential confounders. In the IHDS surveys, all household-related factors were based on interview with the head of the household, and child-specific factors were based on interview with the child’s mother.

### Data analysis

The study did not have a prespecified analysis plan. The distribution of the outcome variables and potential effect modifiers or confounders with the exposure of early childhood nutritional status were tabulated empirically and after accounting for survey design weights and clustering of multiple children within a household. Weighted means and standard error were used to describe the distribution of continuous variables. Crude differences in the proportion and means of these variables across early childhood nutrition indicators was assessed using chi-squared and *t* test for categorical and continuous variables, respectively. The association of early childhood undernutrition with preadolescent physical growth and cognitive achievement were assessed using survey-weighted logistic and ordinal logistic regression, respectively. Effect estimates were adjusted for all potential confounders, except for type of school, age at school enrollment, and absenteeism because these factors might lie in the pathway of child undernutrition influencing cognitive outcomes. The differential association of early childhood undernutrition on preadolescent outcomes based on the child’s sex at different ages, location of residence, caste, household wealth, and highest male and female education level in the household were assessed using separate multivariable logistic regression models that also adjusted for other confounders. We presented the findings as adjusted predicted probabilities across different levels. Adjusted predicted probabilities were calculated by performing inverse logit transformation of beta coefficients of adjusted models based on their sex across different levels of covariates. We used Stata postestimation command of *contrast* to calculate linear polynomial weighted test for differences in predicted probabilities of a given outcome between children. Multicollinearity was assessed among covariates using variance inflation factor (VIF), as described previously in the literature [17,18], and none of the factors had VIF value greater than 5 were performed in Stata MP 15.

### Sensitivity analysis

To account for possible bias from missing data, we performed sensitivity analysis using 2 methods: (1) a selection-based approach, where missingness was predicted using a subset of variables “U,” which were identified using a forward stepwise selection method with significance value of alpha = 0.10; and (2) multiple imputation using chained equations (5 imputations and 25 burn-in iterations) to impute missing values for missing covariates and assessing adequacy of burn-in period by examining stationarity of each chain by the end of burn-in periods from 1 to 30. Survey-weighted regression estimates were derived in both approaches. For multiple imputation, this was achieved by chained equation, and errors were estimated using the Monte Carlo method [19,20].

### Ethics

The IRB at the University of Massachusetts Medical School reviewed the protocol and exempted it from the full committee review because the publicly available data contained no personal identifiable information on survey participants.

## Results

Among the 7,868 children eligible for this study, more than one-half (57.3%) were undernourished in the first 5 years of life (**Table 1**). Two-thirds of the children lived in rural regions, and there were more male than female children. Nearly half of the children belonged to households where none of the females attended any formal schooling (48.1%). Early childhood undernutrition was associated with nearly all potential confounders and effect modifiers. **Table 2** presents the distribution of physical and cognitive outcomes among 8- to 11-year-old children. Almost half of the children could read paragraphs (52.4%) and subtract (45.5%).

**Table 1:** Summary statistics of eligible children from the IHDS according to their early childhood nutritional status.

	Overall		Undernourished^a^		
	*N*	w-%^b^	*n*	w-%^b^	*p* ^c^
Number of children	7,868	100.0	4,334	57.3	–
**Location**	Missing = 0				
Urban	2,354	24.3	1,143	21.4	<0.001
Rural	5,514	75.6	3,191	78.6	
**Sex**	Missing = 0				
Male	4,199	52.8	2,305	52.0	0.32
Female	3,669	47.2	2,029	48.0	
**HH size**	Missing = 0				
4 or less	1,576	19.3	819	18.8	0.62
5 to 6 people	2,700	34.1	1,534	34.6	
>6 people	3,592	46.6	1,981	46.6	
**Monthly spending quartile in 2005**	Missing = 3				
1 (lowest)	2,000	26.7	1,287	31.0	<0.001
2	2,003	27.7	1,169	29.0	
3	1,932	24.7	1,008	23.0	
4 (highest)	1,930	20.8	867	17.0	
**Highest male education in 2005**	Missing = 158				
None	1,667	23.7	1,064	27.8	<0.001
Primary (1 to 5 years)	1,211	16.4	741	18.1	
Secondary (6 to 10)	3,059	37.1	1,637	35.5	
Higher secondary+ (>10)	1,773	22.8	803	18.6	
**Highest female education in 2005**	Missing = 40				
None	3,428	48.1	2,133	54.0	<0.001
Primary (0 to 5 years)	1,165	13.9	688	14.4	
Secondary (6 to 10)	2,282	27.6	1,100	23.9	
Higher secondary+ (>10)	953	10.4	394	7.7	
**Caste**	Missing = 83				
General	2,026	24.2	976	21.3	<0.001
Other backwards class	3,277	44.2	1,826	44.0	
Scheduled caste/tribe	2,482	31.6	1,490	34.7	
**Religion**	Missing = 0				
Hindu	6,282	81.1	3,483	81.1	0.96
Non-Hindu	1,586	18.9	851	18.9	
**Child age in 2011 to 2012**	Missing = 0				
8	1,798	22.8	910	21.2	0.04
9	1,979	25.0	1,124	25.8	
10	2,345	30.0	1,335	30.7	
11	1,746	22.5	965	22.3	
**Age in years at school enrollment**	Missing = 576				
Weighted mean, SE	5.01	1.33	5.09	0.04	<0.001
**School type**	Missing = 228				
Government	4,794	65.8	2,784	69.3	<0.001
Private	2,846	34.2	1,411	30.7	
**Years of schooling**	Missing = 2				
Weighted mean, SE	3.26	0.04	3.15	0.05	<0.001
**Days/month absent from school**	Missing = 31				
Weighted mean, SE	3.69	0.10	3.56	4.79	0.01

^a^ Assessed using CIAF (if child was either stunted, wasted, or underweight).

^b^ Weighted %.

^c^ Differences in weighted proportion or means by undernutrition status.

CIAF, Composite Index of Anthropometric Failure; IHDS, India Human Development Survey; HH, household; SE, standard error.

**Table 2:** Distribution of preadolescent cognitive and physical outcomes of eligible children from the IHDS eligible for this study.

Exposure variable	Overall		Undernourished^a^		
	*N*	w-%^b^	*n*	w-%^b^	*p* ^c^
Number of children	7,868	100.0	4,334	57.3	–
**Reading level in 2011 to 2012**	Missing = 1,434				
Cannot read	667	11.8	416	13.8	<0.001
Recognizes letters	891	14.6	572	16.5	
Reads words	1,294	21.3	742	22.2	
Reads paragraphs	1,249	19.5	665	19.3	
Reads stories	2,333	32.9	1,142	28.2	
**Math level in 2011 to 2012**	Missing = 1,454				
Does not recognize numbers	990	17.1	640	20.5	<0.001
Recognizes numbers	2,262	37.4	1,377	40.6	
Subtracts	2,001	29.5	996	26.1	
Divides	1,161	16.0	518	12.8	
**BMI category in 2011 to 2012**	Missing = 1,239				
Grade 3 thinness (<16 kg/m^2^)	576	8.1	375	9.5	<0.001
Grade 2 thinness (16 to <17)	776	11.2	487	12.4	
Grade 1 thinness (17 to <18.5)	1,759	27.1	1,051	30.1	
Normal weight (18.5 to <25)	2,937	45.0	1,473	40.6	
Overweight (25 to <30)	364	5.3	158	4.3	
Obese (30 or higher)	217	3.3	115	3.1	
**Sex-adjusted HAZ**^**d**^ **in 2011 to 2012**	Missing = 988				
<−2 SD below mean	5,098	72.6	2,740	67.7	<0.001
≥−2 below mean	1,782	27.4	1,243	32.4	

^a^ Assessed using CIAF (if child was either stunted, wasted, or underweight).

^b^ Weighted percentage.

^c^ Differences in weighted proportion or means by undernutrition status.

^d^ HAZ.

BMI, body mass index; CIAF, Composite Index of Anthropometric Failure; HAZ, height-for-age z-score; IHDS, India Human Development Survey; SD, standard deviation.

The association of early childhood undernutrition and child sex with short stature (HAZ < −2) and thinness (BMI < 18.5 kg/m^2^) during the preadolescent period is presented in **Table 3**. After adjusting for confounders, early childhood undernutrition was associated with a 73% and 52% increase in the odds of having short stature or having BMI less than 18.5, respectively. Similarly, children who were undernourished during early childhood had 24% and 28% lesser odds of achieving a higher category of cognitive achievement in reading and arithmetic, respectively, during their preadolescent period (**Table 4**). Sensitivity analysis performed to quantitatively estimate the bias introduce due to missing data revealed that our findings were robust (**S1–S3 Tables**), with the magnitude of association increasing slightly among imputed dataset.

**Table 3:** Results of bivariate and multivariable survey-weighted logistic regression for the association between children’s nutritional status in first 5 years and their physical growth at age 8 to 11 as measured based on data collected during the 2 waves of IHDS (2004 to 2005 and 2011 to 2012).

	Unadjusted			Adjusted^a^		
**(A) Short stature**^**b**^ **in 2011 to 2012**	**OR**	**LCI**	**UCI**	**OR**	**LCI**	**UCI**
Undernourished^c^ in 2004 to 2005 (ref: No)	*n* = 6,880			*n* = 6,650		
Yes	1.85	1.56	2.19	1.73	1.45	2.06
Child sex (ref: male)	*n* = 6,880			*n* = 6,650		
Female	1.37	1.17	1.60	1.37	1.17	1.60
**(B) BMI < 18.5 kg/m**^**2**^ **in 2011 to 2012**	OR	LCI	UCI	OR	LCI	UCI
Undernourished^c^ in 2004 to 2005 (ref: No)	*n* = 6,629			*n* = 6,419		
Yes	1.66	1.46	1.89	1.52	1.33	1.73
Child sex (ref: male)	*n* = 6,629			*n* = 6,419		
Female	1.02	0.89	1.17	0.99	0.86	1.14

^a^ All 3 models were adjusted for the same covariates: state focus classification, rural or urban residence, household size, expenditure tertile, highest male and female education, caste, religion, and child age in 2012.

^b^ Short stature as defined by HAZ below −2.

^c^ Assessed using CIAF (if child was either stunted, wasted, or underweight).

BMI, body mass index; CIAF, Composite Index of Anthropometric Failure; HAZ, height-for-age z-score; IHDS, India Human Development Survey; LCI, lower confidence interval; OR, odds ratio; UCI, upper confidence interval.

**Table 4:** Results of bivariate and multivariable survey-weighted ordinal logistic regression models for association between children’s nutritional status in first 5 years and their cognitive ability at age 8 to 11 based on data collected in the 2 panels of IHDS.

	Unadjusted			Adjusted^a^		
**(A) Reading score**^**b**^ **in 2011 to 2012**	**CumOR**	**LCI**	**UCI**	**CumOR**	**LCI**	**UCI**
Undernourished^d^ in 2004 to 2005 (ref: No)	*n* = 6,434			*n* = 6,153		
Yes	0.64	0.55	0.73	0.76	0.66	0.87
Child sex (ref: male)	*n* = 6,434			*n* = 6,153		
Female	0.96	0.85	1.09	1.01	0.97	1.14
**(B) Mathematics score**^**c**^ **in 2011 to 2012**	CumOR	LCI	UCI	CumOR	LCI	UCI
Undernourished^d^ in 2004 to 2005 (ref: No)	*n* = 6,414			*n* = 6,153		
Yes	0.57	0.50	0.65	0.72	0.63	0.82
Child sex (ref: male)	*n* = 6,414			*n* = 6,153		
Female	0.79	0.69	0.90	0.79	0.69	0.90

^a^ All 3 models were adjusted for the same covariates: state focus classification, rural or urban residence, child sex, household size, expenditure tertile, highest male and female education, caste, religion, type of school, and child age in 2012.

^b^ 0 to 4 score: 0—cannot read, reads letters, reads words, and reads paragraphs and 4—reads stories.

^c^ 0 to 3 score: 0—does not recognize numbers, can count, and can subtract and 3—can divide.

^d^ Assessed using CIAF (if child was either stunted, wasted, or underweight).

CIAF, Composite Index of Anthropometric Failure; cumOR, cumulative odds ratio; IHDS, India Human Development Survey; LCI, lower confidence interval; UCI, upper confidence interval.

**Fig 2:**
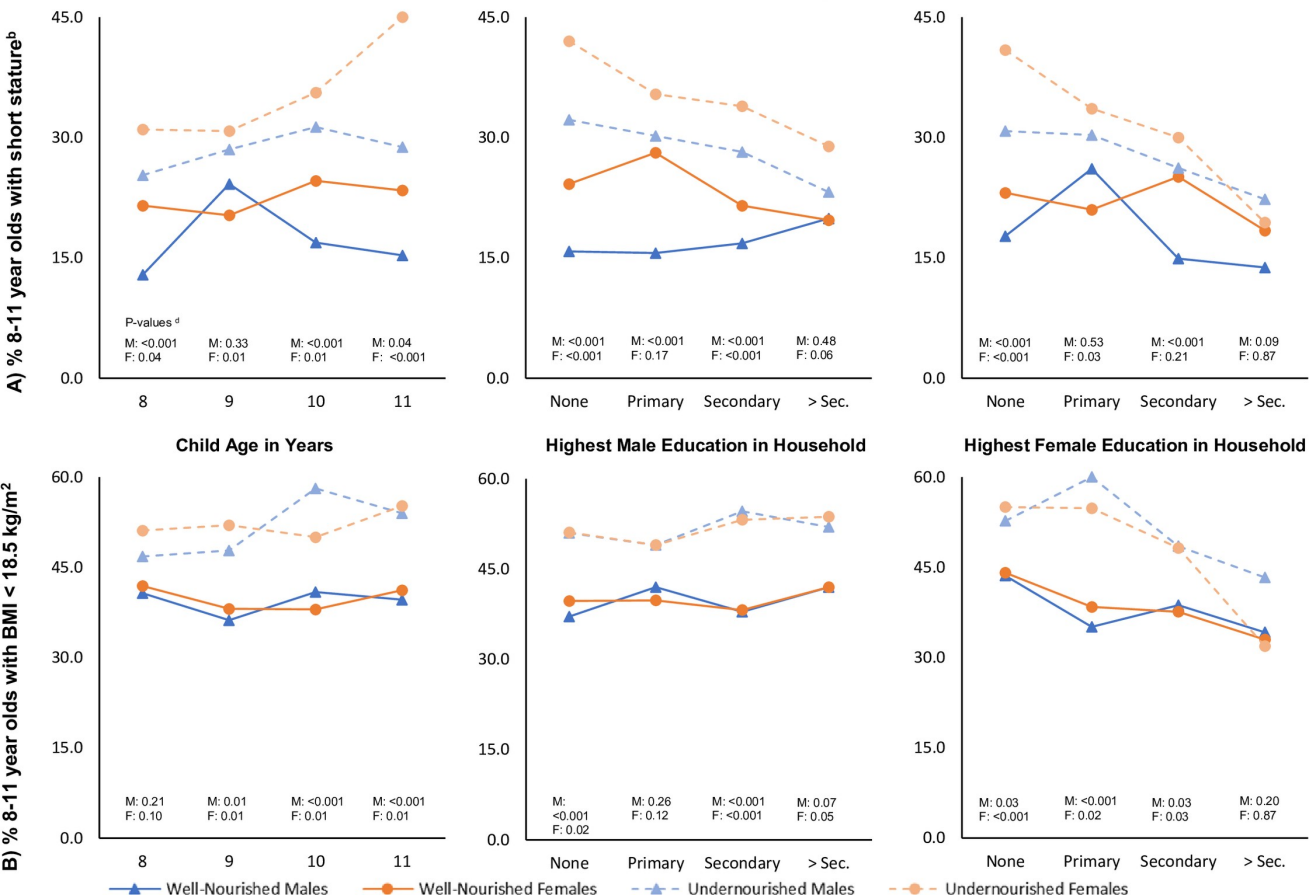
Adjusted predicted probabilities^a^ for subgroups of 8- to 11-year-old Indian children having **(A)** short stature^b^ or **(B)** BMI below 18.5 kg/m^2^ based on their nutritional status^c^ during early childhood (0 to 5 years) and gender. BMI, body mass index.

The association between early childhood and short stature in preadolescence differed by child sex, child age, and highest male and female education within the household (**Fig 2A**). The adjusted predicted probability of short stature for undernourished female children increased from 31% among 8 year olds to 45% among 11 year olds. By contrast, male undernourished children do not experience a consistently increased risk of having short stature with increased age. Increasing levels of highest male and female education within the household mitigated the disadvantage of early childhood undernutrition on short stature, especially among female children living in households with higher level of female education. There were no discernible differences for the probability of short stature between undernourished and well-nourished female children belonging to households where the highest level of female education was secondary or higher. This observation of higher female education associated with mitigation of the relationship between early childhood undernutrition and short stature during preadolescent period was also observed for BMI (**Fig 2B**).

**Fig 3:**
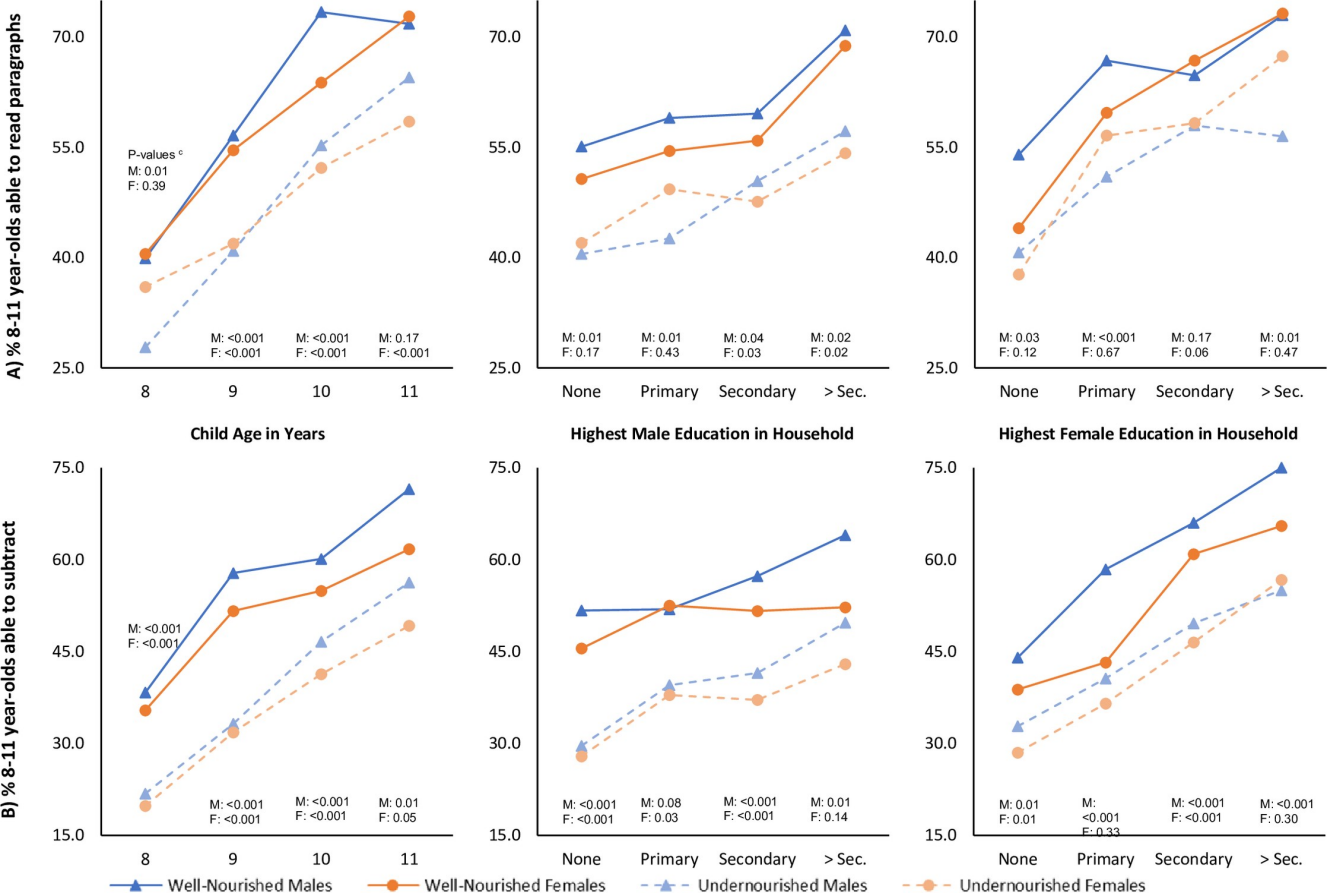
Adjusted predicted probabilities^a^ for subgroups of 8- to 11-year-old Indian children being able to **(A)** read paragraphs or **(B)** subtract based on their nutritional status^b^ during early childhood (0 to 5 years) and gender.

The ability of 8- to 11-year-old children to read paragraphs increased with age, but this improvement differed by sex and early childhood nutritional status (**Fig 3A**). Among 8 year olds, there was no statistically significant difference in the ability to read paragraphs between well-nourished and undernourished female children (*p* = 0.39), while undernourished male children were significantly less likely to be able to read in comparison to well-nourished male children (*p* = 0.01). However, this pattern was reversed among 11 year olds such that early childhood undernourishment was associated with a lower probability to read paragraphs among female (*p* < 0.001) but not male children (*p* = 0.17). Increasing levels of male education within the household improves the probability of male and female children to be able to read paragraphs, but the disadvantage of early childhood undernutrition persists. In contrast, increasing levels of female education within the household was associated with a dampening of the disadvantage of early childhood CIAF on reading ability among female children.

Similar to reading ability, the ability to subtract increases with age and higher levels of male and female education within the household (**Fig 3B**). However, there is a greater increase for male children than female children. The association of early childhood undernutrition with decreased ability to subtract during preadolescent years is observed across all subgroups regardless of child sex, except for households where at least 1 female has attended more than 10 years of schooling (*p* = 0.30). Results of additional analyses for the association of early childhood undernutrition with preadolescent physical and cognitive outcomes based on the child’s sex and location of residence, household caste, and household wealth are presented in **S1 and S2 Figs**. These results suggest that the association of early childhood undernutrition and adverse preadolescent outcomes were stronger among children who lived in rural regions, belonged to scheduled caste or tribe households, and were in a lower quartile of household wealth.

## Discussion

In this analysis of the first nationally representative panel dataset from India, we found that early childhood undernutrition was adversely associated with a number of physical and cognitive outcomes during the preadolescent period. Our findings suggest that CIAF as an indicator of early childhood undernutrition is consistently associated with the physical and cognitive outcomes later in their childhood. We observed that the association between early childhood undernutrition and anthropometric status persists throughout the preadolescent period, and 11-year-old female children with a history of undernourishment face an even greater risk of having short stature in comparison to 8 year olds. Not surprisingly, we observed that children’s ability to read paragraphs and subtract improves with age. However, our results reveal that the magnitude of those improvements differ by child sex and early childhood undernutrition status. Lastly and most importantly, our findings suggest that having higher female education levels within the household was associated with a mitigation of the disadvantage of early childhood undernutrition for physical and cognitive outcomes during preadolescence, especially among female children. By contrast, higher male education levels within the household were not associated with a similar mitigation of the disadvantage of early childhood undernutrition for male and female children.

Female children in India experience the most rapid height growth associated with transition to adolescence between the ages of 8 and 11 years old [21]. Therefore, our observation of an increasing probability of short stature over time among female children with a history of early childhood undernutrition is of concern because it might lead to short stature in adulthood. A community-based longitudinal investigation of child height based on nutritional status also supports our finding that the disadvantage of undernutrition among females does not manifest until later ages [22]. Female children in India are breastfed less than male children [23]. They also have lower dietary diversity than male children, and this disparity widens during the early adolescent period [24]. On average, parents spend less time with their female children in comparison with their male offspring in India [23]. Female children are more likely to terminate schooling early and get co-opted by parents to contribute to household and babysitting activities [25]. These observations might underlie our finding of sex-based disparities in short stature that are observed to be associated with early childhood nutritional status.

Our results indicate that female sex and early childhood undernutrition are associated with poorer reading and arithmetic ability in later years. This finding is corroborated by other studies, which have found that early childhood undernutrition is not only associated with deficits in academic achievement, but also deficits in psychosocial competencies that are predictive of adult educational and productivity outcomes [26,27]. Because maternal height, maternal BMI, and maternal education are the most important predictors of undernutrition in the first 5 years [7,28], our findings highlight the intergenerational nature of undernutrition. We observed that association between child undernutrition and preadolescent outcomes is more prominent among females. Subsequently, children of these women, who are more likely to be short statured, thin, and less educated, face an increased risk of undernutrition. Our finding that higher levels of female education within the household mitigates the association between early childhood undernutrition and adverse outcomes among female preadolescents may be key to overcoming this vicious cycle.

It is important to understand why higher female education in the household may be protective in this setting. Mothers in India are expected to fulfill household, occupational, and child-rearing responsibilities while receiving limited family support, placing considerable constraints on their ability to effectively care for their children [25]. Within this context, several interrelated mechanisms may help explain the importance of female education. First, increased female education may allow women in the household to negotiate family dynamics and experience greater autonomy in decision-making [29]. Increased maternal autonomy is associated with improved nutrition and vaccination status among Indian children [30]. Second, increased maternal education is associated with better recognition of the nutritional status of their children in India and might facilitate a timely intervention [31]. Third, presence of a female with a higher education level within a household is associated with more equitable preferences for gender roles and may reduce the expectation of female children to participate in household chores [25]. Fourth, women of reproductive age in India who have higher levels of education are better prepared to cope with stress, mitigating risk of developing depressive symptoms [32]. Data from India and other countries show that maternal depressive symptoms significantly increases the risk of early child undernutrition and development [33].

Nutrition-sensitive interventions to promote child growth and development are likely to continue having a less than desirable effect if the children’s primary advocate, their mother, continues to carry a disproportionate share of responsibilities and face gender-based discrimination. Female disadvantage in India begins even prior to birth in the form of sex selective abortions [34]. The ratio of male to female children under the age of 5 has increased from 1.04 in 1992 to 1.09 in 2015 (authors’ calculation of the National Family and Health Survey data) [35]. Considering that evidence suggests that higher female education leads to economic and social empowerment of women and a more equitable male to female ratio at birth [36], promotion of education for women should assume a central role in Indian public health policy making.

The findings of our study should be considered in the context of its limitations. First, measurement of exposure was missing or implausible for a subset of participants; similarly, outcome data were missing for a portion of the children who were successfully followed up 8 years later. We performed multiple imputation to account for the missing data and found that our findings did not change in a major way. Additionally, we were reassured by the finding that our exposure variable, undernutrition, measured through this dataset is comparable to other nationally representative datasets [37]. We measured exposure at a single time point, increasing the likelihood for time-varying misclassification. It is possible that a child classified as well-nourished may become undernourished. The likely consequence of such misclassification is an underestimation of the effect of undernutrition on outcomes during preadolescence. Because we were interested in examining the consequences of ever being undernourished during early childhood, the reverse scenario of misclassifying a child as undernourished, who later recovers within the first 5 years, is of a lesser concern for the validity of our findings. Results from smaller, community-based studies that measured undernutrition at multiple time points during early childhood corroborate our findings, suggesting that the findings of our study are unlikely to be due to classification error [38]. As with all observational studies, we cannot rule out the possibility of residual confounding. However, the consistent pattern of physical and cognitive deficits based on child’s sex and poor early nutrition suggest that any variable which might be able to diminish the association observed is likely to be closely linked with the constructs of gender-based discrimination and disadvantages of early childhood nutrition.

In conclusion, our findings, based on the best available nationally representative data, offer important insights regarding early child undernutrition and its association with physical and cognitive outcomes during preadolescent period in India and highlight possible solutions to overcome this intransigent crisis. We demonstrate that improved female education within the household is important not only for preventing child undernutrition but also for mitigating its association with physical and cognitive underachievement. It is possible that the protection conferred by increased female education have downstream effects because preadolescent physical and cognitive status are strong predictors of adult stature and education level. Thus, elevation of women’s status through improved female education should lie at the core of national level programs and initiatives to improve maternal and child health.

## Supporting information

S1 RECORD Checklist RECORD, REporting of studies Conducted using Observational Routinely collected Data. (DOCX)

S1 Table Weighted proportion of characteristics for participants in the dataset with measured and unmeasured exposure and outcome variables. (DOCX)

S2 Table Association between early child nutritional status and physical outcomes in preadolescent period based on (a) complete case analysis, (b) modeling missingness as a covariate, (c) multiple imputation, and (d) continuous covariates using survey-weighted logistic regression.
(DOCX)

S3 Table Association between early child nutritional status and cognitive outcomes in preadolescent period based on (a) complete case analysis, (b) modeling missingness as a covariate, (c) multiple imputation, and (d) continuous covariates using survey-weighted logistic regression.
(DOCX)

S1 Fig Adjusted predicted probabilities^a^ for subgroups of 8- to 11-year-old Indian children having **(A)** short stature^b^ or **(B)** BMI below 18.5 kg/m^2^ based on their nutritional status^c^ during early childhood (0 to 5 years) and gender. BMI, body mass index.
(TIF)

S2 Fig Adjusted predicted probabilities^a^ for subgroups of 8- to 11-year-old Indian children being able to **(A)** read paragraphs or **(B)** subtract based on their nutritional status^b^ during early childhood (0 to 5 years) and gender.
(TIF)

## Abbreviations

ASER: Annual Status of Education Report
BMI: body mass index
CI: confidence interval
CIAF: Composite Index of Anthropometric Failure
cumOR: cumulative odds ratio
HAZ: height-for-age z-score
ICPSR: Inter-university Consortium for Political and Social Research
IHDS: India Human Development Survey
OR: odds ratio
RECORD: REporting of studies Conducted using Observational Routinely collected Data
VIF: variance inflation factor
WAZ: weight-for-age z-score
WHO: World Health Organization
WHZ: weight-for-height z-score
