# Supplementary material for: Early childhood undernutrition, preadolescent physical growth, and cognitive achievement in India: A population-based cohort study
Source: PLoS Med. 2021 Oct 27;18(10):e1003838. doi: 10.1371/journal.pmed.1003838 (PMC8580255; doi:10.1371/journal.pmed.1003838)
Supplement: S1 Table — (DOCX) [file pmed.1003838.s002.docx]

| Supplementary Table 1: Weighted proportion of characteristics for participants in the dataset with measured and unmeasured exposure and outcome variables | | | | | |
| --- | --- | --- | --- | --- | --- |
|  | Overall | Analytical sample | Missing Exposure | | Missing Outcome |
| Number of children | 24,314 | 7,868 | 10,314 | | 6,132 |
| **Location** |  | | |  | |
| Urban | 28.0 | 29.9 | 22.7 | | 19.1 |
| Rural | 72.0 | 70.1 | 77.3 | | 80.9 |
| **Sex** |  | | |  | |
| Male | 51.9 | 53.4 | 52.5 | | 50.5 |
| Female | 48.1 | 46.6 | 47.5 | | 49.5 |
| **Household (HH) Size** |  | | |  | |
| 4 or less | 18.4 | 19.3 | 20.0 | | 15.0 |
| 5-6 people | 32.6 | 34.1 | 31.2 | | 33.0 |
| > 6 people | 49.0 | 46.6 | 48.8 | | 52.0 |
| **Monthly Spending Quartile in 2005** |  | | |  | |
| 1 (Lowest) | 27.0 | 26.7 | 26.0 | | 28.9 |
| 2 | 27.3 | 27.7 | 24.2 | | 31.2 |
| 3 | 24.7 | 24.7 | 25.0 | | 24.3 |
| 4 (Highest) | 21.0 | 20.8 | 24.8 | | 15.6 |
| **Highest Male Education in 2005** |  | | |  | |
| None | 23.6 | 23.7 | 21.0 | | 27.3 |
| Primary (1-5 years) | 17.0 | 16.4 | 15.9 | | 19.4 |
| Secondary (6-10) | 37.5 | 37.1 | 38.6 | | 36.1 |
| Higher Secondary+ (>10) | 21.9 | 22.8 | 24.5 | | 17.2 |
| **Highest Female Education in 2005** |  | | |  | |
| None | 49.2 | 48.0 | 44.5 | | 57.2 |
| Primary (0-5 years) | 13.8 | 13.9 | 13.6 | | 13.9 |
| Secondary (6-10) | 27.0 | 27.6 | 29.3 | | 22.9 |
| Higher Secondary+ (>10) | 10.0 | 10.4 | 12.6 | | 6.0 |
| **Caste** |  | | |  | |
| General | 22.5 | 24.2 | 22.3 | | 20.5 |
| Other Backwards Caste | 45.2 | 44.2 | 45.8 | | 46.0 |
| Scheduled Caste/Tribe | 32.4 | 31.6 | 31.9 | | 33.5 |
| **Religion** |  | | |  | |
| Hindu | 81.6 | 81.1 | 80.2 | | 83.2 |
| Non-Hindu | 18.4 | 18.9 | 19.8 | | 16.8 |
