# Supplementary material for: Early childhood undernutrition, preadolescent physical growth, and cognitive achievement in India: A population-based cohort study
Source: PLoS Med. 2021 Oct 27;18(10):e1003838. doi: 10.1371/journal.pmed.1003838 (PMC8580255; doi:10.1371/journal.pmed.1003838)
Supplement: S3 Table — Association between early child nutritional status and cognitive outcomes in preadolescent period based on (a) complete case analysis, (b) modeling missingness as a covariate, (c) multiple imputation, and (d) continuous covariates using survey-weighted logistic regression. (DOCX) [file pmed.1003838.s004.docx]

| Supplementary Table 3: Association between early child nutritional status and cognitive outcomes in pre-adolescent period based on a) complete case analysis b) modeling missingness as a covariate c) multiple imputation and d) continuous covariates using survey-weighted logistic regression | | | | | | | | | | | | |
| --- | --- | --- | --- | --- | --- | --- | --- | --- | --- | --- | --- | --- |
|  | Complete case | | | Missingness^d^ | | | Imputed^e^ | | | Continuous^f^ | | |
| **A) Reading score^b^ in 2011-12** | Cum. OR | LCI | UCI | Cum. OR | LCI | UCI | Cum. OR | LCI | UCI | Cum. OR | LCI | UCI |
| Undernourished^c^ in 2004-05 (ref: No) | n = 6,153 | | | n = 6,153 | | | n = 7,868 | | | n = 6,153 | | |
| Yes | 0.76 | 0.66 | 0.87 | 0.75 | 0.65 | 0.86 | 0.76 | 0.67 | 0.88 | 0.77 | 0.67 | 0.88 |
| Child sex (ref: male) | n = 6,153 | | | n = 6,153 | | | n = 7,868 | | | n = 6,153 | | |
| Female | 1.01 | 0.97 | 1.14 | 1.01 | 0.89 | 1.14 | 1.04 | 0.91 | 1.18 | 1.04 | 0.92 | 1.17 |
| **B) Mathematics score^c^ in 2011-12** | Cum. OR | LCI | UCI | Cum. OR | LCI | UCI | Cum. OR | LCI | UCI | Cum. OR | LCI | UCI |
| Undernourished ^c^ in 2004-05 (ref: No) | n = 6,153 | | | n = 6,153 | | | n = 7,868 | | | n = 6,153 | | |
| Yes | 0.72 | 0.63 | 0.82 | 0.72 | 0.63 | 0.83 | 0.73 | 0.64 | 0.83 | 0.71 | 0.62 | 0.81 |
| Child sex (ref: male) | n = 6,153 | | | n = 6,153 | | | n = 7,868 | | | n = 6,153 | | |
| Female | 0.79 | 0.69 | 0.90 | 0.79 | 0.69 | 0.90 | 0.82 | 0.72 | 0.94 | 0.82 | 0.72 | 0.93 |
| OR: Odds Ratio; LCI: Lower Confidence Interval; UCI: Upper Confidence Interval;  a: all models were adjusted for the same covariates: state focus classification, rural or urban residence, household size, expenditure tertile, highest male and female education, caste, religion, and child age in 2012. b: short stature as defined by height-for-age z score below -2 c: Assessed using Composite Index of Anthropometric Failure (if child was either stunted, wasted, or underweight)  d: Missingness model used a selection method to identify probability of having missing data and using this variable as a confounder in the final model.  e: Survey weighted regression estimates from multiple imputation chained equation. Variables included in the imputation were: expenditure tertile, household size, sex, rural/urban location, state, district, population sampling unit, survey weight, number of children, number of married members, household assets, income per capita, age of male head of family, age of female head of family, religion, education level of household family members  f: covariates were considered as continuous | | | | | | | | | | | | |
