# Supplementary figures and images for: Early childhood undernutrition, preadolescent physical growth, and cognitive achievement in India: A population-based cohort study
Source: PLoS Med. 2021 Oct 27;18(10):e1003838. doi: 10.1371/journal.pmed.1003838 (PMC8580255; doi:10.1371/journal.pmed.1003838)

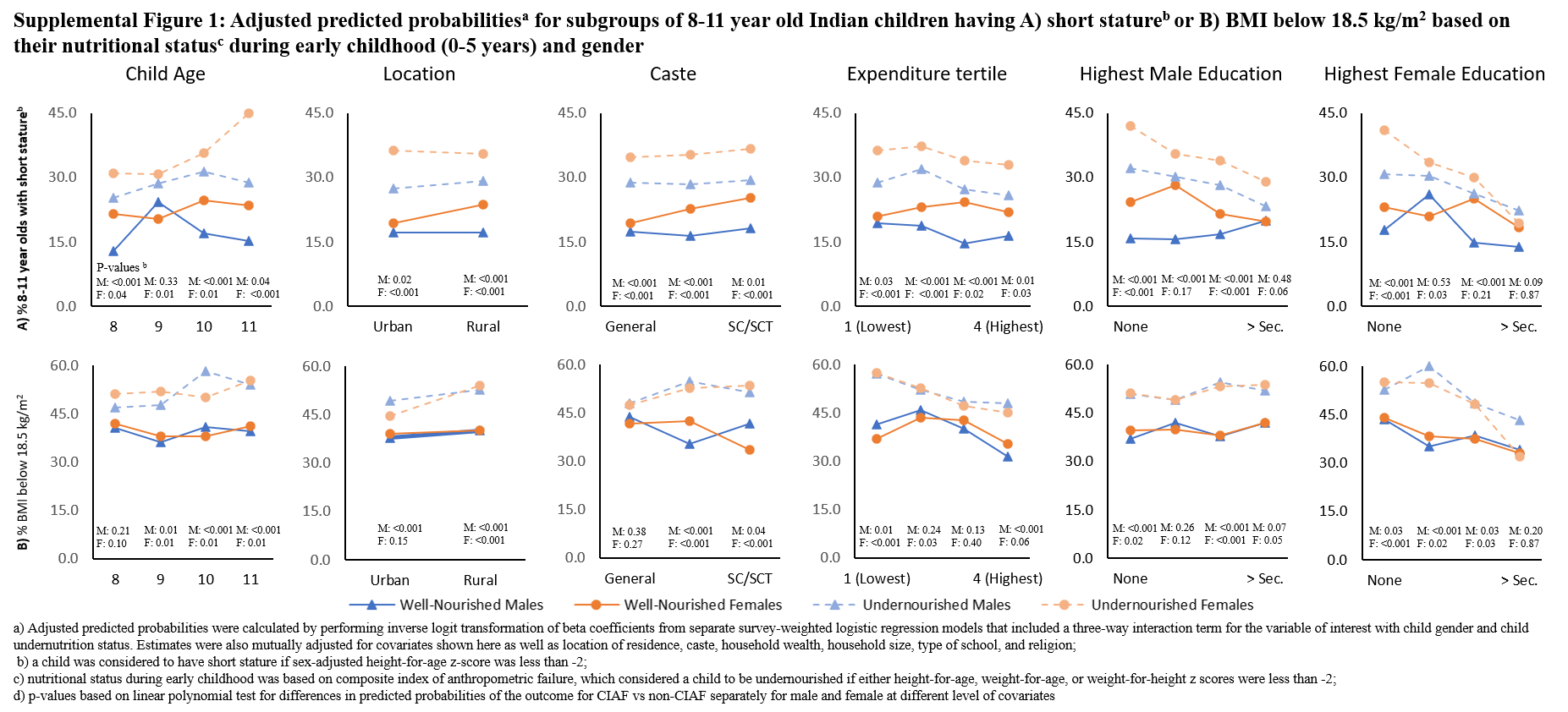

Supplement: S1 Fig — Adjusted predicted probabilitiesa for subgroups of 8- to 11-year-old Indian children having (A) short statureb or (B) BMI below 18.5 kg/m2 based on their nutritional statusc during early childhood (0 to 5 years) and gender. BMI, body mass index. (TIF) [file pmed.1003838.s005.tif]

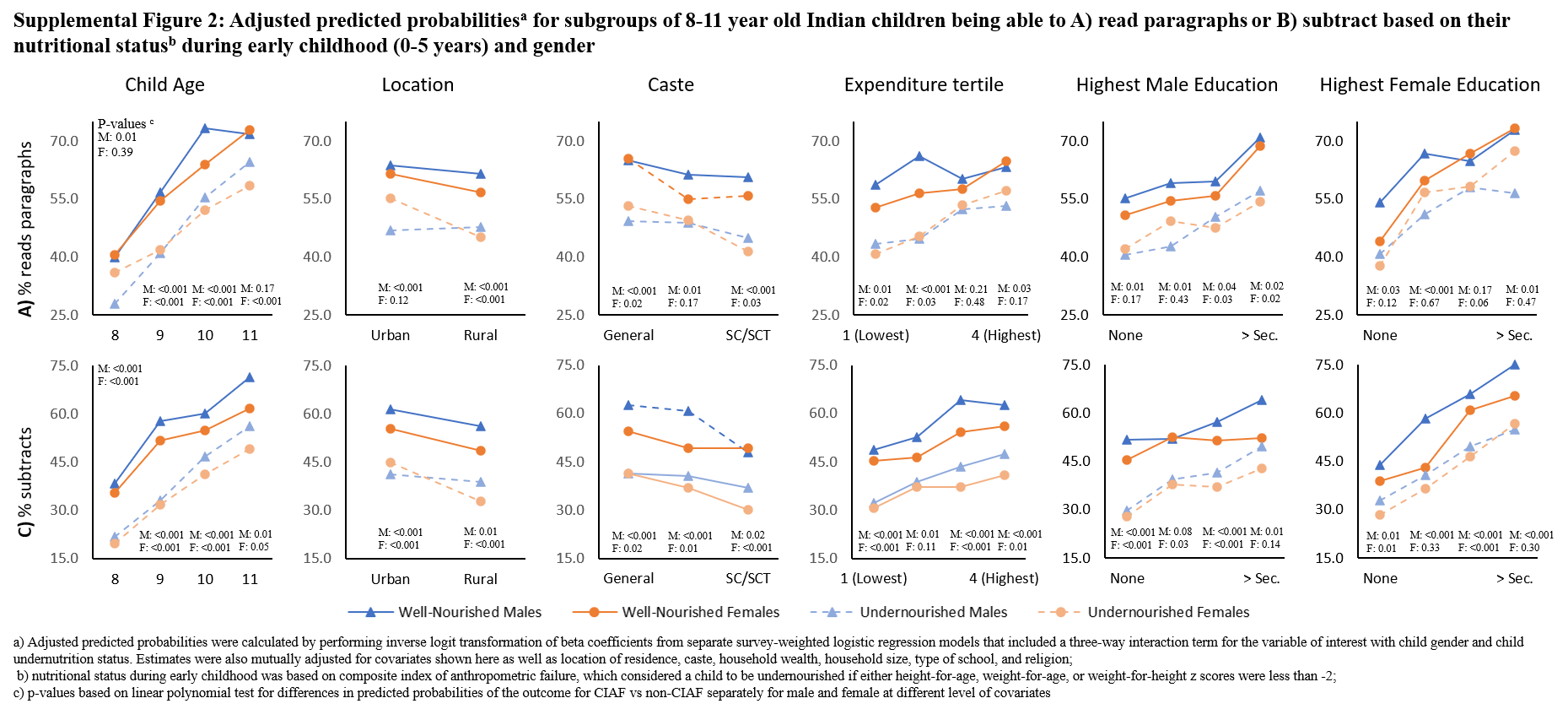

Supplement: S2 Fig — Adjusted predicted probabilitiesa for subgroups of 8- to 11-year-old Indian children being able to (A) read paragraphs or (B) subtract based on their nutritional statusb during early childhood (0 to 5 years) and gender. (TIF) [file pmed.1003838.s006.tif]
